# A species checklist of the subgenus Culicoides (Avaritia) in China, with a description of a new species (Diptera, Ceratopogonidae)

**DOI:** 10.3897/zookeys.706.13535

**Published:** 2017-10-04

**Authors:** Qiong Qiong Chang, Xiao Hong Jiang, Guo Ping Liu, Xiao Fei Li, Xiao Hui Hou

**Affiliations:** 1 Zunyi Medical University, Zunyi 563000, Guizhou Province, China; 2 Center for Disease Control and Prevention of Shenyang PLA Command, Shenyang 110034, Liaoning Province, China

**Keywords:** Biting midges, Ceratopogonidae, checklist, *Culicoides*, subgenus *Avaritia*

## Abstract

A checklist of the subgenus Culicoides (Avaritia Fox) (Diptera: Ceratopogonidae: *Culicoides*) in China, currently including 57 species, is provided. Their full citations, more detailed locations of the type locality, and distribution of each species by province, and/or state of each species are also provided. Culicoides (Avaritia) fenggangensis Liu & Hou, **sp. n.** is described and illustrated, based on both male and female specimens from China. The new species is compared with its similar congeners, C. (A.) comparis Liu & Yu, 2005 and C. (A.) dentiformis McDonald & Lu, 1972.

## Introduction

Biting midges of the genus *Culicoides* Latreille (Diptera, Ceratopogonidae) are found everywhere in the world and females are the smallest of insect vectors (Mellor et al. 2000). Some species of *Culicoides* spread disease in humans and livestock as vectors of arboviruses, such as bluetongue virus (BTV), Schmallenberg virus (SBV), and others (Borkent 2005), which leads to direct economic costs for agriculture (Gibbens 2012; Velthuis et al. 2010). In China, Bluetongue has been recorded from several provinces in south, such as Yunnan, Hubei, Anhui, Sichuan, Shanxi and so on (Zhang et al. 2015), and the vector *Culicoides* of BTV mainly distributed in the south region of north latitude 40° (Zhang et al. 2014). Therefore, members of this genus have great international significance and have attracted more scientific attention in recent years. The genus *Culicoides*, currently with a total number of species of 1415 in the world, 1368 extant species and 47 fossil species (Borkent, 2016), has 348 species in China. Many of the important vectors are in the subgenus Culicoides (Avaritia Fox). The purpose of this paper is to provide a checklist of this subgenus in China, and describe and illustrate Culicoides (A.) fenggangensis sp. n.

## Materials and methods

The specimens were collected with light traps near households in the mountains of Fenggang County of Guizhou Province. For microscopic observation, specimens were preserved in 100% ethanol and then slide-mounted in Canada balsam following the technique described by Yu et al. (2005). Diagnostic features were microphotographed using an Imaging System of Upright Research Microscope adapted to a microscope (Nikon Eclipse Ni-E) and a Digital System of Large depth-of-field 3D Digital Microscope (Keyence VHX-1000C), and Photoshop CS4 was used to obtain the final images. Morphological terms are from the chapter on Ceratopogonidae by Yu et al. (2005). Terms of structures specific to *Culicoides* follow those described by Santarém et al. (2014) and Han et al. (2017). Measurements of wings, flagellar segments, palpus, and legs are given in millimeters, and measurements of spermathecae are given in microns. Meristic information is presented as ranges of values, followed by mean and sample size. The type specimens are deposited in Insect Collection of Zunyi Medical University, Guizhou Province, China.

## Taxonomy

### Checklist of the subgenus Avaritia in China in alphabetical order

**Subgenus Avaritia Fox, 1955**

*Avaritia* Fox, 1955: 218.

**Type species**. *Ceratopogon obsoletus* Meigen, 1818.

**Culicoides (Avaritia) abchazicus Dzhafarov, 1964**

*Culicoides abchazicus* Dzhafarov, 1964: 263; Liu et al. 2011: 385. Type Locality: Georgia.

**Distribution.** China (Liaoning); Georgia.

**Culicoides (Avaritia) actoni Smith, 1929**

*Culicoides actoni* Smith, 1929: 255; McDonald and Lu 1972: 411; Lee 1988: 29; Yu et al. 2005: 907. Type locality: India.

*Culicoides okumensis* Arnaud, 1956: 119. Type locality: Japan.

*Culicoides imperceptus* Das Gupta, 1962a: 538. Type locality: India.

**Distribution.** China (Heilongjiang, Jiangsu, Anhui, Fujian, Taiwan, Shandong, Hubei, Guangdong, Guangxi, Hainan, Sichuan, Yunnan, Tibet, Shaanxi, Hunan); India, Indonesia, Malaysia, Philippines, Vietnam, Japan, Thailand.

**Culicoides (Avaritia) albifascia Tokunaga, 1937**

*Culicoides albifascia* Tokunaga, 1937: 319; Lee 1978: 28; Yu et al. 2005: 908. Type locality: China: Taiwan.

**Distribution.** China (Heilongjiang, Tibet, Sichuan, Yunnan, Taiwan).

**Culicoides (Avaritia) bawanglingensis Yu, Wang & Chen, 2012**

*Culicoides bawanglingensis* Yu, Wang & Chen, in Wang et al., 2012: 283. Type Locality: China: Hainan, Bawangling.

**Distribution.** China (Hainan).

**Culicoides (Avaritia) brevipalpis Delfinado, 1961**

*Culicoides brevipalpis* Delfinado, 1961: 654; Yu et al. 2005: 910. Type locality: Philippines.

**Distribution.** China (Taiwan, Guangdong, Hainan, Yunnan); Australia, Indonesia, Malaysia, Japan, Sri Lanka, Thailand, Philippines.

**Culicoides (Avaritia) brevitarsis Kieffer, 1917**

*Culicoides brevitarsis* Kieffer, 1917: 187; Yu et al. 2005: 911. Type locality: Australia.

*Culicoides robertsi* Lee & Reye, 1953: 386. Type locality: Australia.

*Culicoides radicitus* Delfinado, 1961: 657. Type locality: Philippines.

*Culicoides superfulvus* Das Gupta, 1962b: 253. Type locality: India.

**Distribution.** China (Anhui, Taiwan, Hainan); Australia, Philippines, India, Laos, Malaysia.

**Culicoides (Avaritia) bubalus Delfinado, 1961**

*Culicoides bubalus* Delfinado, 1961: 658; Yu et al. 2005: 913. Type locality: The Philippines.

**Distribution.** China (Taiwan), Philippines.

**Culicoides (Avaritia) chiopterus (Meigen), 1830**

*Ceratopogon chiopterus* Meigen, 1830: 263. Type locality: Europe.

*Ceratopogon amoenus* Winnertz, 1852: 35. Type locality: Germany.

*Monohelea similis* Goetghebuer, 1927: 203. Type locality: Belgium.

*Culicoides dobyi* Callot & Kremer, 1969: 610. Type locality: France.

**Distribution.** China (Hebei, Inner Mongolia, Liaoning, Jilin, Heilongjiang, Tibet); Germany, France, Belgium, Britain, Russia.

**Culicoides (Avaritia) clavipalpis Mukerji, 1931**

*Culicoides clavipalpis* Mukerji, 1931: 1052; McDonald and Lu 1972: 403; Yu et al. 2005: 916. Type locality: India.

*Culicoides candidus* Sen & Das Gupta, 1959: 620. Type locality: India.

**Distribution.** China (Jiangsu, Fujian, Shandong, Hainan, Sichuan); India, Indonesia, Laos, Malaysia, Philippines, Thailand.

**Culicoides (Avaritia) comparis Liu & Yu, 2005**

*Culicoides comparis* Liu & Yu, in Yu et al., 2005: 917. Type locality: China: Tibet, Nielamu.

**Distribution.** China (Tibet).

**Culicoides (Avaritia) conaensis Liu & Yu, 1990**

*Culicoides conaensis* Liu & Yu, 1990: 19; Yu et al. 2005: 919. Type locality: China: Tibet, Cuona.

**Distribution.** China (Tibet).

**Culicoides (Avaritia) dentiformis McDonald & Lu, 1972**

*Culicoides dentiformis* McDonald & Lu, 1972: 403; Yu et al. 2005: 921. Type locality: China: Taiwan.

**Distribution.** China (Taiwan).

**Culicoides (Avaritia) elongatus Chu & Liu, 1978**

*Culicoides elongatus* Chu & Liu, 1978: 83; Yu et al. 2005: 922. Type locality: China: Yunnan, Mengla.

**Distribution.** China (Fujian, Yunnan).

**Culicoides (Avaritia) fenggangensis Liu & Hou, sp. n.**

*Culicoides fenggangensis* Liu & Hou, sp. n., this paper. Type locality: China: Guizhou, Fenggang.

**Distribution.** China (Guizhou).

**Culicoides (Avaritia) filicinus Gornostaeva & Gachegova, 1972**

*Culicoides filicinus* Gornostaeva & Gachegova, 1972: 522; Yu et al. 2005: 924. Type locality: Russia.

**Distribution.** China (Tibet, Heilongjiang); Russia.

**Culicoides (Avaritia) gaponus Yu, 1982**

*Culicoides gaponus* Yu, 1982: 202; Yu et al. 2005: 925. Type locality: China: Hainan, Diaoluoshan.

**Distribution.** China (Hainan).

**Culicoides (Avaritia) holcus Lee, 1980**

*Culicoides holcus* Lee, 1980: 85; Yu et al. 2005: 927. Type locality: China: Yunnan.

**Distribution.** China (Yunnan).

**Culicoides (Avaritia) hui Wirth & Hubert, 1961**

*Culicoides hui* Wirth & Hubert, 1961: 16; Yu et al. 2005: 928. Type locality: China: Taiwan.

**Distribution.** China (Taiwan, Guangdong, Hainan, Yunnan, Hunan); Indonesia, Laos, Malaysia.

**Culicoides (Avaritia) imicola Kieffer, 1913**

*Culicoides imicola* Kieffer, 1913: 11; Yu et al. 2005: 930. Type locality: Kenya.

*Culicoides pallidipennis* Carter, Ingram & Macfie, 1920: 265. Type locality: Ghana.

*Culicoides iraqensis* Khalaf, 1957: 343. Type locality: Iraq.

*Culicoides minutus* Sen & Das Gupta, 1959: 622. Type locality: India.

*Culicoides pseudoturgidus* Das Gupta, 1962a: 537. Type locality: India.

**Distribution.** China (Hainan); Wide spread in Africa, the Middle and Far East, India, Laos, Sri Lanka, Vietnam.

**Culicoides (Avaritia) incertus Yu & Zhang, 1988**

*Culicoides incertus* Yu & Zhang, in Yu, 1988: 136; Yu et al. 2005: 931. Type locality: China: Tibet.

**Distribution.** China (Tibet).

**Culicoides (Avaritia) innoxius Sen & Das Gupta, 1959**

*Culicoides innoxius* Sen & Das Gupta, 1959: 626; Yu et al. 2005: 933. Type locality: India.

**Distribution.** China (Hainan); India, Cambodia, Indonesia, Laos, Malaysia, Sri Lanka, Thailand.

**Culicoides (Avaritia) insignipennis Macfie, 1937**

*Culicoides insignipennis* Macfie, 1937b: 469; Lee 1988: 65; Yu et al. 2005: 935. Type locality: Malaysia.

**Distribution.** China (Fujian, Taiwan, Yunnan, Hunan); Malaysia, Brunei, Indonesia, Laos, Philippines, Singapore, Thailand.

**Culicoides (Avaritia) iphthimus Zhou & Lee, 1984**

*Culicoides iphthimus* Zhou & Lee, 1984: 295; Yu et al. 2005: 936. Type locality: China: Chongqing.

**Distribution.** China (Chongqing).

**Culicoides (Avaritia) jacobsoni Macfie, 1934**

*Culicoides jacobsoni* Macfie, 1934: 215; Yu 1982: 48. Yu et al. 2005: 938. Type locality: Indonesia.

*Culicoides buckleyi* Macfie, 1937a: 117. Type locality: Malaysia.

*Culicoides kitaokai* Tokunaga, 1955: 6. Type locality: Japan.

*Culicoides unisetiferus* Tokunaga, 1959: 236. Type locality: Papua New Guinea.

**Distribution.** China (Fujian, Taiwan, Guangdong, Guangxi, Hainan, Yunnan, Tibet, Hunan); Indonesia, Malaysia, Japan, New Guinea, Philippines, Thailand.

**Culicoides (Avaritia) kepongensis Lee, 1988**

*Culicoides kepongensis* Lee, 1988: 69; Wirth and Hubert 1989: 346; Yu et al. 2005: 940. Type locality: China; Malaysia.

**Distribution.** China (Fujian, Taiwan); Malaysia, Laos, Thailand.

**Culicoides (Avaritia) kinabaluensis Wirth & Hubert, 1989**

*Culicoides kinabaluensis* Wirth & Hubert, 1989: 211; Yu et al. 2005: 941. Type locality: Indonesia.

**Distribution.** China (Hainan).

**Culicoides (Avaritia) lansangensis Howarth, 1985**

*Culicoides lansangensis* Howarth, 1985: 58; Liu et al. 1996: 38; Yu et al. 2005: 942. Type locality: Laos.

**Distribution.** China (Guangdong, Hainan, Hunan).

**Culicoides (Avaritia) lengi Yu & Liu, 1990**

*Culicoides lengi* Yu & Liu, 1990: 10; Yu et al. 2005: 944. Type locality: China: Guangdong, Shixing.

**Distribution.** China (Guangdong).

**Culicoides (Avaritia) liui Wirth & Hubert, 1961**

*Culicoides liui* Wirth & Hubert, 1961: 20; Yu et al. 2005: 946. Type locality: China: Taiwan.

**Distribution.** China (Taiwan, Yunnan); Indonesia, Laos, Malaysia, Philippines, Thailand.

**Culicoides (Avaritia) longirostris Qu & Wang, 1994**

*Culicoides longirostris* Qu & Wang, 1994: 486; Yu et al. 2005: 947. Type locality: China: Tibet.

**Distribution.** China (Tibet).

**Culicoides (Avaritia) malayae Macfie, 1937**

*Culicoides malayae* Macfie, 1937b: 471; Yu 1982: 190; Yu et al. 2005: 949. Type locality: Malaysia.

**Distribution.** China (Fujian, Taiwan, Guangdong, Guangxi, Hainan, Yunnan); Indonesia, Malaysia, Philippines, Thailand.

**Culicoides (Avaritia) mamaensis Lee, 1979**

*Culicoides mamaensis* Lee, 1979: 101; Yu et al. 2005: 951. Type locality: China: Tibet, Cuona.

**Distribution.** China (Sichuan, Tibet).

**Culicoides (Avaritia) motoensis Lee, 1978**

*Culicoides motoensis* Lee, 1978: 75; Yu et al. 2005: 952. Type locality: China: Tibet, Motuo.

**Distribution.** China (Tibet).

**Culicoides (Avaritia) nielamensis Liu & Deng, 2000**

*Culicoides nielamensis* Liu & Deng, 2000: 246; Yu et al. 2005: 954. Type locality: China: Tibet, Nielamu.

**Distribution.** China (Tibet).

**Culicoides (Avaritia) nigritus Fei & Lee, 1984**

*Culicoides nigritus* Fei & Lee, 1984: 345; Yu et al. 2005: 955. Type locality: China: Inner Mongolia, Linxi.

**Distribution.** China (Inner Mongolia).

**Culicoides (Avaritia) nujiangensis Liu, 1990**

*Culicoides nujiangensis* Liu, 1990: 59; Yu et al. 2005: 956. Type locality: China: Yunnan, Nujiang.

**Distribution.** China (Yunnan).

**Culicoides (Avaritia) obsoletus (Meigen), 1818**

*Ceratopogon obsoletus* Meigen, 1818: 76. Type locality: Europe.

*Ceratopogon varius* Winnertz, 1852: 35. Type locality: Germany.

*Ceratopogon yezoensis* Matsumura, 1911: 60. Type locality: Russia.

*Culicoides obscuripes* Santos Abreu, 1918: 297. Type locality: Spain.

*Culicoides lacteinervis* Kieffer, 1919a: 47. Type locality: Slovak Republic, Ukraine.

*Culicoides rivicola* Kieffer, 1921: 56. Type locality: Germany.

*Culicoides clavatus* Kieffer, 1921: 56. Type locality: Germany.

*Culicoides heterocerus* Kieffer, 1921: 57. Type locality: Germany.

*Culicoides pegobius* Kieffer, 1922: 235. Type locality: Germany.

*Culicoides kabyliensis* Kieffer, 1922: 505. Type locality: Algeria.

*Culicoides concitus* Kieffer, 1922: 71. Type locality: Germany.

*Culicoides intermedius* Okada, 1941: 22. Type locality: Japan.

*Culicoides sintrensis* Cambournac, 1956: 591. Type locality: Portugal.

*Culicoides seimi* Shevchenko, 1967: 173. Type locality: Ukraine.

**Distribution.** China (Shanxi, Inner Mongolia, Liaoning, Jilin, Heilongjiang, Fujian, Shandong, Sichuan, Chongqing, Yunnan, Xinjiang, Gansu, Tibet); wide distribution in Palaearctic Region, Britain, Germany, Russia, Canary Islands, Algeria, Japan, Portugal, Slovakia, Ukraine.

**Culicoides (Avaritia) orestes Wirth & Hubert, 1989**

*Culicoides orestes* Wirth & Hubert, 1989: 222; Yu et al. 2005: 959. Type locality: Malaysia.

**Distribution.** China (Hainan); Malaysia.

**Culicoides (Avaritia) orientalis Macfie, 1932**

*Culicoides orientalis* Macfie, 1932: 490; Lee 1978: 83; Yu et al. 2005: 961. Type locality: Malaysia.

*Culicoides nayabazari* Das Gupta, 1963: 35. Type locality: India.

**Distribution.** China (Fujian, Taiwan, Hainan, Sichuan, Yunnan, Tibet, Hunan); Malaysia, India, Indonesia, Philippines, Thailand, Vietnam, the Solomon Islands.

**Culicoides (Avaritia) palauensis Tokunaga, 1959**

*Culicoides palauensis* Tokunaga, in Tokunaga & Murachi, 1959: 348; Yu 1982: 57; Yu et al. 2005: 963. Type locality: USA.

**Distribution.** China (Guangdong, Hainan, Yunnan); Oceania.

**Culicoides (Avaritia) pastus Kitaoka, 1980**

*Culicoides pastus* Kitaoka, 1980: 11; Lee 1988: 91; Yu et al. 2005: 964. Type locality: Japan.

**Distribution.** China (Sichuan, Yunnan); Japan.

**Culicoides (Avaritia) pelius Liu & Yu, 1990**

*Culicoides pelius* Liu & Yu, 1990: 23; Liu and Deng 2000: 245; Yu et al. 2005: 966. Type locality: China: Tibet, Cuona.

**Distribution.** China (Tibet).

**Culicoides (Avaritia) peregrinus Kieffer, 1910**

*Culicoides peregrinus* Kieffer, 1910: 191; Yu et al. 2005: 967. Type locality: India.

*Culicoides judicandus* Bezzi, 1916: 8. Type locality: Philippines.

*Culicoides esmoneti* Salm, 1917b: 136. Type locality: Indonesia.

*Culicoides philippinensis* Kieffer, 1921: 564. Type locality: Philippines.

*Culicoides assamensis* Smith & Swaminath, 1932: 183. Type locality: India.

*Culicoides quadratus* Tokunaga, 1951: 108. Type locality: Indonesia.

**Distribution.** China (Hebei, Inner Mongolia, Liaoning, Jiangsu, Fujian, Taiwan, Jiangxi, Henan, Guangdong, Guangxi, Hainan); India, Philippines, Indonesia.

**Culicoides (Avaritia) qionghaiensis Yu & Liu, 1990**

*Culicoides qionghaiensis* Yu & Liu, 1990: 4; Yu et al. 2005: 969. Type locality: China: Sichuan, Xichang.

**Distribution.** China (Sichuan).

**Culicoides (Avaritia) ruiliensis Lee, 1980**

*Culicoides ruiliensis* Lee, 1980: 86; Yu et al. 2005: 971. Type locality: China: Yunnan.

**Distribution.** China (Yunnan).

**Culicoides (Avaritia) scoticus Downes & Kettle, 1952**

*Culicoides scoticus* Downes & Kettle, 1952: 65; Yu et al. 2005: 972. Type locality: Great Britain.

**Distribution.** China (Tibet); Great Britain, France.

**Culicoides (Avaritia) sinanoensis Tokunaga, 1937**

*Culicoides sinanoensis* Tokunaga, 1937: 331; Yu et al. 2005: 973. Type locality: Japan.

*Culicoides obsoletiformis* Amosova, 1957: 233. Type locality: Russia.

**Distribution.** China (Liaoning, Jilin, Heilongjiang, Yunnan, Shaanxi); Japan, Russia.

**Culicoides (Avaritia) suiyangensis Hou, Han, Lv & Jiang, 2014**

*Culicoides suiyangensis* Hou, Han, Lv & Jiang, 2014: 98. Type Locality: China: Guizhou, Suiyang.

**Distribution.** China (Guizhou).

**Culicoides (Avaritia) sumatrae Macfie, 1934**

*Culicoides sumatrae* Macfie, 1934: 190; Yu et al. 2005: 975. Type locality: Malaysia.

*Culicoides amamiensis* Tokunaga, 1937: 325. Type locality: Japan.

*Culicoides kagiensis* Tokunaga, 1937: 327. Type locality: Taiwan.

*Culicoides ohmorii* Takahashi, 1958: 113. Type locality: Japan.

*Culicoides assimilis* Delfinado, 1961: 660. Type locality: Philippines.

**Distribution.** China (Fujian, Taiwan, Guangdong, Guangxi, Hainan, Yunnan, Tibet); Malaysia, Japan, Philippines.

**Culicoides (Avaritia) suzukii Kitaoka, 1973**

*Culicoides suzukii* Kitaoka, 1973: 212; Lee 1988: 107; Yu et al. 2005: 977. Type locality: Japan.

**Distribution.** China (Taiwan, Yunnan); Japan.

**Culicoides (Avaritia) tainanus Kieffer, 1916**

*Culicoides tainanus* Kieffer, 1916: 114; Yu et al. 2005: 979. Type locality: China: Taiwan.

*Ceratopogon maculatus* Shiraki, 1913: 294. Type locality: China: Taiwan.

*Culicoides kii* Tokunaga, 1937: 284. Type locality: Japan.

*Culicoides sigaensis* Tokunaga, 1937: 322. Type locality: Japan.

*Culicoides kyotoensis* Tokunaga, 1937: 329. Type locality: Japan.

*Culicoides suborientalis* Tokunaga, 1951: 106. Type locality: Indonesia.

**Distribution.** China (Fujian, Hainan, Yunnan, Shaanxi, Shandong); Indonesia, Japan, Laos, Malaysia, Philippines, Thailand, Vietnam.

**Culicoides (Avaritia) tibetensis Chu, 1977**

*Culicoides tibetensis* Chu, 1977: 102; Yu et al. 2005: 980. Type locality: China: Tibet.

**Distribution.** China (Sichuan, Tibet).

**Culicoides (Avaritia) trimaculatus McDonald & Lu, 1972**

*Culicoides trimaculatus* McDonald & Lu, 1972: 415; Yu et al. 2005: 982. Type locality: China: Taiwan.

**Distribution.** China (Taiwan).

**Culicoides (Avaritia) wadai Kitaoka, 1980**

*Culicoides wadai* Kitaoka, 1980: 14; Yu et al. 2005: 984. Type locality: China: Taiwan.

**Distribution.** China (Fujian, Guangxi, Taiwan).

**Culicoides (Avaritia) wandashanensis Wang & Liu, 1999**

*Culicoides wandashanensis* Wang & Liu, 1999: 328; Yu et al. 2005: 985. Type locality: China: Heilongjiang, Raohe.

**Distribution.** China (Heilongjiang).

**Culicoides (Avaritia) yamii Lien, Lin & Weng, 1998**

*Culicoides yamii* Lien, Lin & Weng, 1998: 57; Yu et al. 2005: 987. Type locality: China: Taiwan, Lanyu.

**Distribution.** China (Taiwan).

**Culicoides (Avaritia) yuchihensis Lien, Lin & Weng, 1998**

*Culicoides yuchihensis* Lien, Lin & Weng, 1998: 58; Yu et al. 2005: 988. Type locality: China: Taiwan.

**Distribution.** China (Taiwan).

#### Culicoides (Avaritia) fenggangensis

Taxon classificationAnimaliaDipteraCeratopogonidae

Liu & Hou sp. n.

http://zoobank.org/52A53C15-0F18-4040-B83C-B678BD2E9A66

Figs 1 , 2 , 3

##### Diagnosis.

Male: only species of *Culicoides* in China with the following combination of features: the 3^rd^ segment of the palpus is slender, PR 3.11; the apex of 9^th^ tergite has lateral processes; parameres with apical portion elongate, bent abruptly; aedeagus nearly triangular, with a long ovoid process at its apex. Female: only species of *Culicoides* in China with the following combination of features: cell m_2_ with four sparsely distributed pale spots; the 3^rd^ segment of the palpus is slender, PR 3.20–3.75.

##### Description.

**Female.** *Head* (Fig. 2a). Brown. Eyes (Fig. 2b) contiguous, abutting medially for length of 1.5 ommatidia, with interfacetal hairs. Antennal pedicel brown; Lengths of antennal flagellomeres in proportion of 19: 14: 14: 15: 15: 15: 16: 17: 25: 25: 25: 31: 47; AR 1.22–1.28 (1.24, n = 3); sensilla coeloconica on flagellomeres 1, 9–13. 3^rd^ segment of palpus slender, slightly swollen at apical 1/3, with small, rounded sensory pit (Fig. 2c–d); PR 3.20–3.75 (3.45, n = 3). Mandible with 14–18 (16, n = 4) teeth (Fig. 2e); Maxilla with 17 teeth (n = 3); P/H ratio 1.04–1.17 (1.11, n = 3).

**Figure 1.**
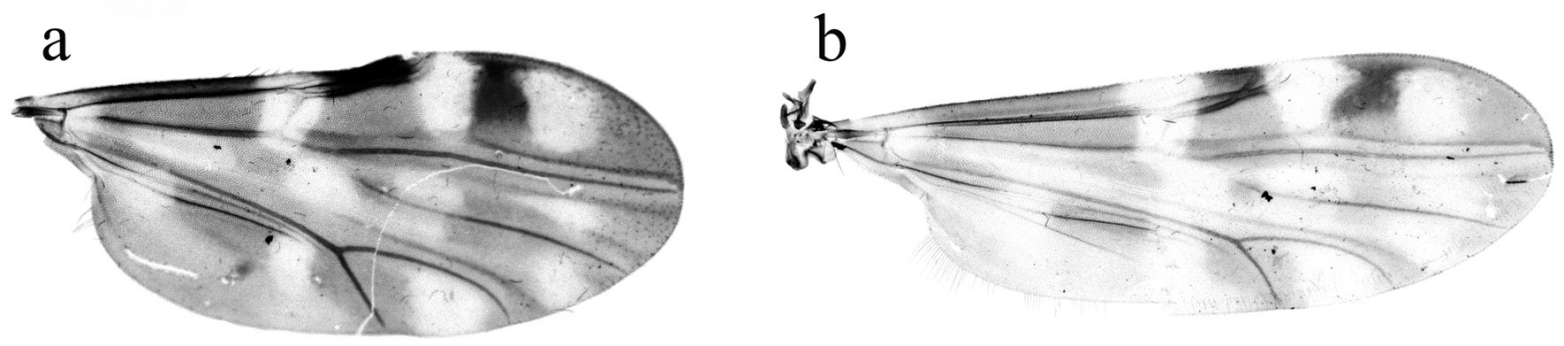
Culicoides (A.) fenggangensis sp. n. Left Wing. **a** Female **b** Male.

**Figure 2.**
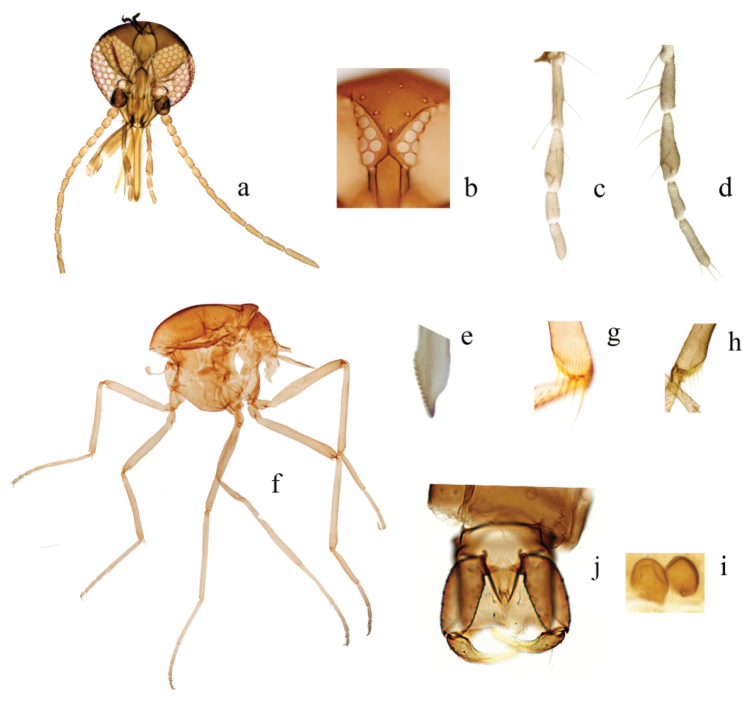
**a–j** Culicoides (A.) fenggangensis sp. n. **a** Head, anterior view **b** Eye contiguous, anterior view **c** Left palpus, anterior view **d** Right palpus, anterior view **e** Mandibular teeth **f** Thorax and legs, lateral view **g** Apex of hind tibia and base of first tarsomere, posterior view. **h** Apex of hind tibia and base of first tarsomere, posterior view. **i** Spermathecae **j** Genitalia, ventral view (Female: **a–c, e–i**; Male: **d, j**).

*Thorax* (Fig. 2f). Dark brown. Scutellum without distinct pattern in slide-mounted specimens. Wing (Fig. 1a) with contrasting pattern of pale, dark spots; distal 1/2 of 1^st^, proximal 1/2 of 2^nd^ radial cell in dark spot; three pale spots near anterior margin: 1^st^ over base of cell r_1_ and r-m crossvein extending from below M_1_ to margin of costa, 2^nd^ over distal of cell r_2_ from dorsal portion of M_1_ to costa, 3^rd^ morphological variation, from dorsal portion of M_1_ to just below costa; cell m_1_ with two separated pale spots: one spot small, ovoid, another large, triangle, far from distal portion of wing; cell m_2_ with four different shapes, sizes pale spots: 1^st^ proximal to CuA, 2^nd^ between medial and mediocubital forks, 3^rd^ and 4^th^ below M_2_, latter reaching wing margin; cua_1_ with a large, ovoid pale spot abutting wing posterior margin; anal cell with two pale spots: proximal pale spot on Cu_2_ and CuA, distal pale spot near mediocubital fork; wing base with faint pale spot on M; macrotrichia sparsely distributed on distal 1/3 of wing, but not in basal cell; wing length 1.45–1.60 (1.52, n = 3) mm, width 0.68–0.73 (0.70, n = 3) mm; CR 0.55–0.59 (0.57, n = 3). TR and F-T of legs (Fig. 2f) are given as Table 1, metatibial distal bristles (Fig. 2g–h) with 5 or 6 spines, 1^st^ spine is longest.

**Table 1.** Tarsal ratios (TR) and measurements of leg segments and tarsomeres from femur to tarsomere 5 (F-T) of all legs of *C. fenggangensis* sp. n. (♀).

Leg	TR	F-T
Foreleg	2.12	85 : 88 : 53 : 25 : 16 : 10 : 11
Midleg	2.31	109 : 114 : 60 : 26 : 16 : 10 : 11
Hindleg	1.73	109 : 115 : 57 : 33 : 17 : 11 : 12

*Abdomen*. Brown. Two subequal-size ovoid spermathecae (Fig. 2i), measuring 65.0–72.5 × 50.0–57.5 (n = 3) μm, 65.0–72.5 × 50.0 (n = 2) μm, slender sclerotized necks with 2.5–7.5 (n = 3) μm; third slender, elongate rudimentary spermatheca, length 20.0–25.0 (n = 3) μm.

**Figure 3.**
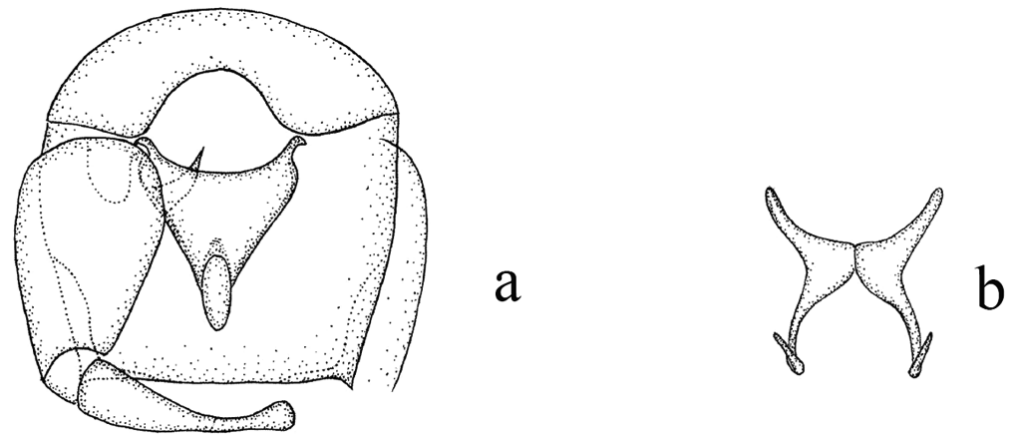
Culicoides (A.) fenggangensis sp. n. **a** Genitalia and aedeagus (parameres absent), ventral view **b** parameres, ventral view.

**Male**. Similar to female with usual sexual differences. Sensilla coeloconica on flagellomeres 1, 11–13; AR 0.87 (n = 1); PR 3.11 (n = 1). Wing with pattern of pale spots as in Fig. 1b, wing length 1.58 (n = 1), width 0.55 (n = 1); CR 0.57 (n = 1). TR and F-T of legs are given as Table 2. Genitalia (Fig. 2j): 9^th^ tergite squarish, distal portion flat with short, conical processes at apicolateral. Ninth sternite with broad, deep, semicircle caudomedian excavation. Gonocoxite twice as long as broad, sclerotized; gonostylus tapering distally, distal portion curved. Parameres (Fig. 3b) separate, contiguous in midportions; each with moderately long, slender basal arm, swollen at base, stem long, slightly curved near base; apical portion tapered, elongate, abruptly bent without lateral fringe of spicules. Aedeagus (Fig. 3a) nearly triangle, basal arms short, unciform, tapering toward end, basal arch low, extending to 1/5 of total length, distal process 1/6 total length, long ovoid process at apex.

**Table 2.** TR and F-T of all legs of *C. fenggangensis* sp. n. (♂).

Leg	TR	F-T
Foreleg	2.67	95 : 92 : 56: 21 : 15 : 8 : 9
Midleg	1.94	115 : 112 : 64 : 33 : 16 : 8 : 9
Hindleg	1.49	117 : 117 : 55 : 37 : 19 : 9 : 10

**Type material.** Holotype female, Chongxin village, Yong’an town, Fenggang county, Zunyi city, Guizhou province, China (28°06'31.49"N, 107°35'57.94"E), 12. IV. 2016, alt. 908m, Qiongqiong Chang col. Paratypes: 2 males and 3 females, same data as holotype.

##### Distribution.

China (Guizhou Province).

##### Etymology.

This species is named in tribute to Fenggang county, where the specimens were collected.

## Taxonomic discussion

Culicoides (Avaritia) fenggangensis sp. n. is very similar to C. (A.) comparis Liu & Yu, 2005 and C. (A.) dentiformis McDonald & Lu, 1972 based on the interfacetal hairs on the eyes and sensilla coeloconica on the flagellomeres. *C. fenggangensis* sp. n. can be distinguished from these two congeners by the number and distribution of pale spots on the wing (cell m_2_ with two and three pale spots respectively in *C. dentiformis* and *C. comparis*) (Yu et al. 2005), elongate and cylindrical third palpus segment (third palpus segment is swollen in *C. comparis* and *C. dentiformis*). Females of *C. fenggangensis* sp. n. have a different wing size (wing length 1.33 mm and width 0.63 mm in *C. comparis* and wing length 1.78 mm and width 0.76 mm in *C. dentiformis*), distribution of the heavy macrotrichia on the wing (respectively distal 4/5 and 1/4 of the wing in *C. comparis* and *C. dentiformis*), they have a more slender 3^rd^ palpus segment compared to the most species of subgenus Avaritia (only 12 species PR > 3.2), PR 3.2–3.75 (respectively PR 2.27 and 2.5 in *C. comparis* and *C. dentiformis*), different size of spermathecae (measuring 45.0 × 40.0 μm in *C. comparis* and 60.0 × 45.0 μm in *C. dentiformis*). Because male of *C. comparis* is unknown, the new species will only compare with *C. dentiformis*. Males of *C. fenggangensis* sp. n. have a different shape and structure of genitalia, with two lateral processes on the distal portion of ninth tergite (without lateral process in *C. dentiformis*), parameres apical portion tapered and abruptly bent (linear in *C. dentiformis*), long ovoid process at apex of aedeagus (diamond-shape process in *C. dentiformis*). Therefore, the distinctive features to separate *C. fenggangensis* sp. n. from others are cell m_1_ and m_2_ with 2 and 4 pale spots respectively.

The male of *C. fenggangensis* keys to C. dentiformis in Yu et al. (2005) where it may be distinguished by the presence of conical apicolateral processes on tergite 9 which are absent in *C. dentiformis*. The female of *C. fenggangensis* keys to the couplet with *C. dentiformis* and *C. comparis* in Yu et al. (2005) where it may be distinguished by the presence of 4 pale spots in cell m_2_ which are absent in *C. dentiformis* and *C. comparis*.

The biogeographical territory of China spans the Palaearctic and Oriental Regions, which results in a rich diversity of biting midge species. The species of the subgenus Culicoides (Avaritia) are distributed in most provinces of China, except Qinghai and Ningxia. There are 15 species distributed in the Palaearctic Region, accounting for 26.3% of the total (*C. abchazicus*, *C. actoni*, *C. albifascia*, *C. chiopterus*, *C. comparis*, *C. incertus*, *C. longirostris*, *C. nielamensis*, *C. nigritus*, *C. obsoletus*, *C. peregrinus*, *C. scoticus*, *C. sinanoensis*, *C. tainanus*, *C. wandashanensis*). There are 49 species present in the Oriental Region, accounting for 86.0% of the total (*C. actoni*, *C. albifascia*, *C. bawanglingensis*, *C. brevipalpis*, *C. brevitarsis*, *C. bubalus*, *C. clavipalpis*, *C. conaensis*, *C. dentiformis*, *C. elongates*, *C. filicinus*, *C. gaponus*, *C. holcus*, *C. hui*, *C. longirostris*, *C. imicola*, *C. innoxius*, *C. insignipennis*, *C. iphthimus*, *C. jacobsoni*, *C. kepongensis*, *C. kinabaluensis*, *C. lansangensis*, *C. lengi*, *C. liui*, *C. malayae*, *C. mamaensis*, *C. motoensis*, *C. nujiangensis*, *C. obsoletus*, *C. orestes*, *C. orientalis*, *C. palauensis*, *C. pastus*, *C. pelius*, *C. peregrinus*, *C. qionghaiensis*, *C. ruiliensis*, *C. sinanoensis*, *C. suiyangensis*, *C. sumatrae*, *C. suzukii*, *C. tainanus*, *C. tibetensis*, *C. trimaculatus*, *C. wadai*, *C. yamii*, *C. yuchihensis*, *C. fenggangensis*). Finally, there are seven species present in both Regions, accounting for 12.3% of the total. This geographical distribution of biting midges in China is consistent with the distribution of other animals (Yan and Yu 1998).

## Supplementary Material

XML Treatment for Culicoides (Avaritia) fenggangensis
